# Winner of the Ronald Melzack - Canadian Journal of Pain Paper of the Year Award for 2025 / Récipiendaire du Prix Ronald Melzack Pour L’Année 2025 Des Articles Parus Dans La Revue Canadienne De La Douleur

**DOI:** 10.1080/24740527.2026.2735618

**Published:** 2026-10-02

**Authors:** Heather Lumsden-Ruegg, Anna Waisman, Joel Katz

**Affiliations:** Canadian Journal of Pain; Canadian Journal of Pain; Canadian Journal of Pain

Every year, the Canadian Journal of Pain and the Canadian Pain Society (CPS) present an award in honor of the late Ronald Melzack, a Canadian Psychologist renowned for his fundamental contributions to pain research and pain management. The award recognizes original articles rated highly with regard to novelty, quality, and potential impact. This year, the Ronald Melzack – *Canadian Journal of Pain* Paper of the Year award was given to Jhalok Ronjan Talukdar, Dena Zeraatkar, Andrew Thomas, and Jason W. Busse from McMaster University and the Canadian Armed Forces Health Services Center for their article titled “An exploration of the increasing prevalence of chronic pain among Canadian veterans: Life After Service Studies 2016 and 2019.” The winning paper was selected by the Editor-in-Chief from a total of 16 original articles published in 2025. The Paper of the Year Award was presented to Dr. Norman Buckley on behalf of the authors by the Chair of the CPS Awards Committee, Dr. Nader Ghasemlou, at the Canadian Pain Society’s 46th Annual Scientific Meeting in Quebec City on April 30, 2026.

Research into chronic pain in military veterans has accelerated in recent years. A higher prevalence of chronic pain has been observed in military veteran populations compared to the general public. A conservative estimate is that ~30% of veterans experience chronic noncancer pain.^1^ Veterans have been identified as a vulnerable population, less likely to report pain and reluctant to seek help, making it imperative to understand veteran-specific factors associated with pain and its management. Recent qualitative work among Canadian veterans with chronic pain has explored veterans’ perceptions of the causes of their pain, the impact of pain on their daily life, and the barriers they face in accessing care.^2^ Military culture led veterans to feel pressure to accept and push through pain, while discouraging or delaying care-seeking to prioritize the mission, their team, and their careers. Veterans noted that seeking care after discharge is complicated by the transition to the civilian healthcare system, limited awareness of available supports, discomfort with seeking care, denied coverage along with other institutional barriers, and limited or no access to specialized pain clinics in the region where they live.^2^

Building on earlier research conducted in Canada, this year’s winning article by Talukdar and colleagues^3^ compared data from 2016 and 2019 obtained from the Life After Service Study (LASS) of Canadian veterans to evaluate changes in chronic pain prevalence between these two timepoints. The study assessed several variables previously associated with chronic pain in veteran populations, including sociodemographic characteristics, military rank and environment, and health-related factors such as BMI and mental illness. Univariate analyses showed that when compared to the 2016 cohort, veterans who completed the study in 2019 were significantly more likely to report chronic pain, pain‑related conditions, anxiety disorders, mood disorders, probable PTSD, and traumatic brain injury. Chronic pain was significantly and positively related to older age, higher BMI, and presence of self-reported mood and anxiety disorders. Although veterans completing the study in 2019 reported approximately a 10% greater prevalence of chronic pain compared to those completing the study in 2016 (41.4% vs. 51.5%), the difference in prevalence was not statistically significant after accounting for differences in reported risk factors. The authors conclude that the higher prevalence in chronic pain observed over time is likely confounded by an increase in reports of comorbid mental health conditions in the 2019 cohort, a finding that we believe deserves serious attention. The authors acknowledge several study limitations and recommend refinements to the Life After Service Study aimed at improving the survey’s definition of chronic pain and reducing selection bias.

The *Journal* Editorial Board and Office and the Canadian Pain Society offer our congratulations to Dr. Talukdar and coauthors for their award-winning article and our thanks for choosing to publish their work in the *Canadian Journal of Pain*. Submit a manuscript to the *Canadian Journal of Pain* now and become eligible for the Ronald Melzack – *Canadian Journal of Pain* Paper of the Year Award for 2026. Next year’s annual scientific meeting of the Canadian Pain Society will be held in Toronto. Who knows? You may find yourself being feted at the awards ceremony and featured in next year’s editorial!
